# Near-infrared emitting AgInTe_2_ and Zn-Ag-In-Te colloidal nanocrystals

**DOI:** 10.1186/s11671-015-0951-y

**Published:** 2015-06-05

**Authors:** Marc-Antoine Langevin, Thomas Pons, Anna M. Ritcey, Claudine Nì. Allen

**Affiliations:** Centre de Recherche sur les Matériaux Avancés (CERMA), Département de Chimie, Université Laval, 1045 Av. de la Médecine, Quebec, QC G1V 0A6 Canada; Centre d’Optique, Photonique et Laser (COPL), Département de Physique, de Génie Physique et d’Optique, Université Laval, 2375 Rue de la Terrasse, Quebec, QC G1V 0A6 Canada; Laboratoire Physique et Étude des Matériaux, ESPCI/CNRS/UPMC UMR8213, 10 Rue Vauquelin, 75005 Paris, France

**Keywords:** Silver indium telluride, I-III-VI semiconductors, Colloidal quantum dots, Near-infrared photoluminescence, Nanocrystal synthesis, Tuneable emission, Ion exchange reaction

## Abstract

The synthesis of AgInTe_2_ nanocrystals emitting between 1095 nm and 1160 nm is presented. Evolution of the Ag:In:Te ratio shows progressive incorporation of In^3+^ in Ag_2_Te, leading to the formation of orthorhombic AgInTe_2_. When zinc is added to the synthesis, the photoluminescence quantum yield reaches 3.4 %.

**Electronic supplementary material:**

The online version of this article (doi:10.1186/s11671-015-0951-y) contains supplementary material, which is available to authorized users.

## Background

In past years, inorganic semiconductor nanocrystals (NCs), including colloidal quantum dots (cQDs), have gained much interest for their use in many applications such as biological imaging, solar cells, analytical chemistry, etc. [1–3]. Although NCs possess interesting properties such as a broad absorption bandwidth and tuneable emission wavelengths, most of them are based on toxic elements such as cadmium, lead, mercury or arsenic, and are hence limited in their industrial use [4–7]. More recently, NCs based on less toxic elements have also been developed [7–12]. I(Ag,Cu)-III(In,Ga)-VI(S,Se,Te)_2_ compounds have been widely studied as bulk materials, principally for their successful use in photovoltaic devices [13]. This has motivated research on nanocrystalline versions in order to generate tuneable electronic and optical properties, especially in the biologically and technologically important near-infrared range of the spectrum [4, 8–10].

In order to harness the full tuning potential of these versatile alloyed nanomaterials, it is necessary to develop new systems and synthetic methods to understand their reactivities. However, with the exception of AgInS_2_, silver-based I-III-VI_2_ NCs have mostly been overlooked. We recently presented a method for the synthesis of near-stoichiometric AgInSe_2_ NCs via thermolysis of an Ag-In-thiolate complex followed by an anion-exchange reaction and indium incorporation [12]. This reaction is possible because the P-Se bonds (315 kJ/mol) of the tributylphosphine selenide (TBP-Se) precursor are weaker than the P-S bonds (403 kJ/mol) [14]. In the present communication, we demonstrate the versatility of this route with the first synthesis of AgInTe_2_ (AIT) NCs. Since the P-Te bonds (218 kJ/mol) are also weaker than the P-S bonds, the anion-exchange route can be employed for the substitution of Te into the NCs [14].

## Methods

In a typical experiment, 0.025 mmol (5.8 mg) of Ag_2_O and 0.05 mmol (14.6 mg) of In(OAc)_3_ were dissolved in 0.25 mL of dodecanethiol (DDT) and 5 mL of octadecene (ODE). After degassing under vacuum at 90 °C for 30 min, 0.25 mL of oleylamine (OLA) was injected in the reaction flask. The solution was then degassed for an additional 30 min and put under N_2_ atmosphere using a standard Schlenk-line technique. The solution was heated to an adequate temperature of 170 °C or 200 °C and 0.2 mL of the Te precursor was injected. This Te precursor was prepared by dissolving 0.55 mmol (70 mg) of Te in 0.25 mL of trioctylphosphine (TOP) and 0.75 mL of ODE. The solution turned dark brown immediately after precursor injection. Injecting TBP instead of TOP in the precursor solution caused instant formation of a black precipitate. The suspensions were allowed to react at constant temperature for 180 min. Aliquots were removed at different reaction times and dispersed in tetrachloroethylene (TCE) for absorption, photoluminescence (PL) and energy dispersive x-ray (EDS) spectroscopic measurements. The NCs were purified by centrifugation in methanol and dispersed in TCE to obtain stable colloidal suspensions. Other solvents such as ethanol, isopropanol and acetone were tested for centrifugation, but they severely quenched the PL emission intensity.

## Results and discussion

**Fig. 1:**
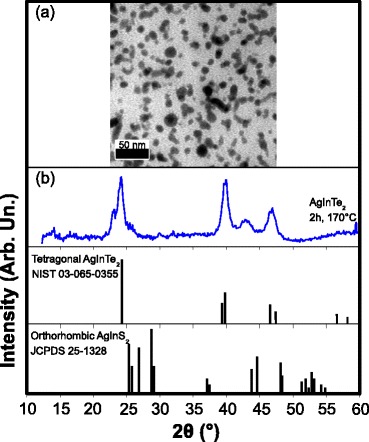
**a** TEM image and **b** XRD pattern of AIT NCs obtained after 120 min of reaction at 170°C. Peaks at 23°, 25.5° and 43° are related to the orthorhombic phase of AgInTe_2_ given their shift relative to AgInS_2_ peaks at smaller angles

The transmission electron microscopy (TEM) image presented in Fig. 1a shows the presence of 10.6 (σ = ±3) nm AIT NCs. Their shape is approximately spherical with a clear trend to form elongated NCs. As shown in Fig. 1b, X-ray diffraction (XRD) analysis confirms that the AIT NCs are mostly in the metastable orthorhombic phase and free of other inorganic compounds after purification. Since no XRD reference pattern is available for orthorhombic AIT, we compare our diffractogram to orthorhombic AgInS_2_, which is shifted towards larger angles due to the smaller size of the sulphur anion [15, 16]. The presence of tetragonal phase cannot be completely excluded because all its main diffraction peaks (24.0°, 39.8° and 46.6°) are also found in the orthorhombic phases.

**Fig. 2:**
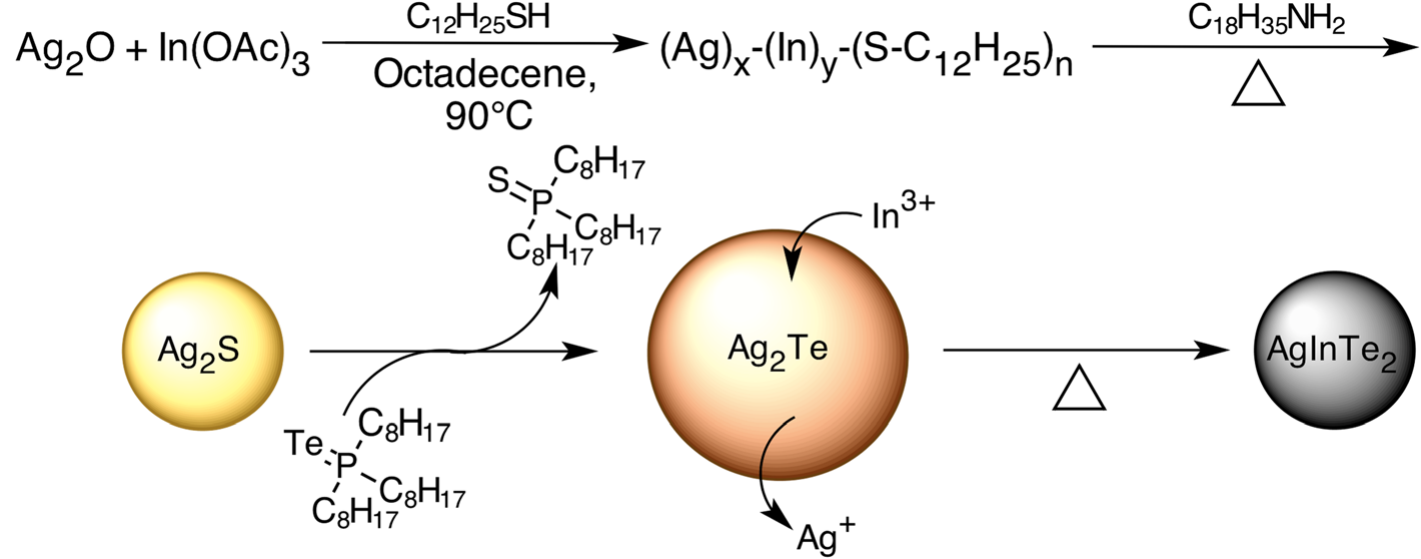
Proposed mechanism for the synthesis of AIT NCs

EDS analysis performed on aliquots taken at different reaction times after TOP-Te injection shows progressive incorporation of In^3+^ in Ag_2_Te NCs as presented in Table 1 and Fig. 2. This is the same reaction mechanism as that proposed for AgInS_2_ and AgInSe_2_ NCs in the orthorhombic phase [12, 17]. Similar incorporation mechanisms were also recently observed for CuInS_2_ NCs [18, 19]. The final AIT NCs are near-stoichiometric and contain, at most, 6.4±0.3 % of sulphur, which can be attributed to the remaining DDT present as a surface ligand. Here, Ag_2_Te NCs are likely to result from an anion-exchange reaction on Ag_2_S formed after thermolysis of the Ag-In-thiolate complex. Even though it was not possible to isolate Ag_2_S NCs at this step of the reaction, an equivalent anion-exchange reaction was attempted on pure ~4 nm Ag_2_S NCs [20] dispersed in ODE with OLA and DDT to demonstrate that this exchange is indeed possible. Ag_2_Te was instantly obtained when TOP-Te was injected at 170 °C as demonstrated by XRD (Additional file 1: Figure S1 of the supporting information).

**Table 1:** Percentage of Ag, In, Te and S in AIT NCs synthesized at 170 °C and 200 °C measured by energy dispersive x-ray spectroscopy

Reaction temperature	Reaction time (min)	% Ag	% In	% Te	% S
170 °C	0.5	57±1	8±1	30±1	5.5±0.6
	1	44±2	18±2	35±2	2.9±0.2
	5	38±3	18.1±0,1	40±2	4.5±0.9
	15	28.9±0.3	24±2	42±2	5.0±0,5
	30	26.8±0.7	23.4±0.9	44.1±0.3	5.6±0.2
	60	24.7±0.3	25.2±0.6	44.4±0.6	5.8±0.1
	120	24±1	27±1	43.6±0.4	6.4±0.3
	360	25±1	30±1	40.1±0.4	3.8±0.2
200 °C	0.5	44±3	20±2	32±1	2.2±0.6
	1	44±2	17±2	34±7	2.6±0.5
	5	34±4	24±3	38±8	3±1
	10	29.0±0.9	34±2	33.4±0.9	3.3±0.8
	15	24±3	36±3	37±1	3.1±0.1
	30	22±1	35±2	39±2	3±1
	120	24.8±0.4	34.3±0.4	37±1	4±1

**Fig. 3:**
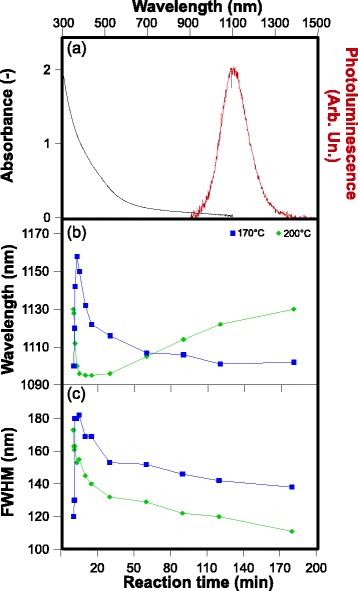
**a** Absorption and emission spectra of AIT NCs after 120 min of reaction at 170 °C. **b** Evolution of the PL peak wavelength and **c** FWHM as a function of the reaction time for AIT NCs synthesized at 170 °C (*blue squares*) and 200 °C (*green diamonds*)

As presented in Fig. 3a, b, this method allows tuning of the AIT NCs’ emission wavelength between 1160 nm and 1095 nm, depending on the reaction temperature and time. At 170 °C, a rapid redshift of the PL peak is observed during the first three minutes of reaction. This is immediately followed by a blueshift, which slows down progressively, reaching a plateau at 1100 nm after 60 min of reaction. The suspension was allowed to react for six more hours, but no significant changes were observed in either the PL spectrum or the composition as monitored by EDS. At 200 °C, we did not observe the initial redshift, possibly because of the faster reaction kinetics. We only observed a rapid blueshift, from 1130 nm to 1095 nm after 5 min, followed by a progressive redshift beginning after 30 min of reaction. NC size distributions are obtained from TEM analysis (Additional file 1: Figure S2) of aliquots prepared under the reaction conditions indicated in Table 2. Their ensemble average and its uncertainty were evaluated by bootstrapping sampling and the results indicate a correlation between NC size and PL emission wavelength, which is graphically confirmed by Additional file 1: Figure S3 in the supporting information. At both reaction temperatures, longer reaction times are also associated to a narrower FWHM of the PL emission spectra (Fig. 3c) and improved relative size dispersion (σ) of NCs (Table 2). To avoid errors that could be induced by NC ripening or other changes during storage, PL measurements and TEM grid preparations were carried out immediately after the synthesis.

**Table 2:** Evolution of the NC size distributions, determined by TEM, and corresponding PL emission wavelengths

Temperature	Time	NC size	σ	Wavelength
(°C)	(min)	(nm)	(nm)	(nm)
170	0.5	10.6±0.4	3	1100
	3	15±1	6	1158
	120	10.7±0.4	3	1101
200	0.2	13±1	4	1130
	30	10.3±0.5	2	1096
	230	12.8±0.5	2	1122

To explain these results, we consider the PL emission mechanisms of I-III-VI_2_ NCs which are complex due to the involvement of defect states and thus depend on the exact stoichiometry for a given crystal structure [21]. These mechanisms fall in two categories: donor-acceptor pair recombination or a transition between one of these defect states and one of the semiconductor bands. If the latter type of mechanisms prevails, it could provide an explanation for the observed spectral shifts and linewidth changes. Finally, we briefly compare the emission characteristics of AgInTe_2_ NCs with those previously reported for AgInSe_2_. In the bulk, the bandgap of the two materials \differs by ~0.3 eV [22]. Both nanomaterials emit in the same range of wavelengths for NCs with a similar ~10 nm size. However, the FWHM is much narrower for NCs with Te (124 nm) instead of Se (357 nm) anions, despite the similar size polydispersity (~25 %) of the two populations [12].

**Fig. 4:**
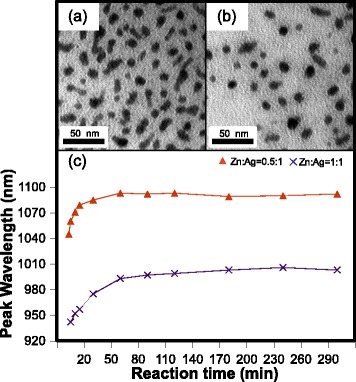
TEM images of Zn-Ag-In-Te NCs with a Zn:Ag ratio of **a** 0.5:1 and **b** 1:1 from aliquots taken after 120 min of reaction. **c** Wavelength of the PL maximum as a function of reaction time for Zn-Ag-In-Te NCs with a Zn:Ag ratio of 0.5:1 (*orange triangles*) and 1:1 (*purple crosses*). All NCs were prepared at a reaction temperature of 170°C

The PL quantum yield (QY) was evaluated relative to ICG (13 % in DMSO) [8]. A maximum PL QY of 0.06 % was measured for AIT NCs synthesized at 170 °C after 120 min reaction. The PL, however, was completely quenched after a single day of storage in ambient conditions, most likely because of surface oxidation often observed for Te-based NCs [23]. Indeed, when kept under a N_2_ atmosphere, the NCs still emitted light after 2 months. Previous studies have shown that it is possible to increase the PL QY of I-III-VI_2_ NCs by incorporating zinc [24]. Therefore, starting from our protocol for AIT NCs synthesized at 170 °C, we added either 0.025 mmol (5.5 mg) or 0.05 mmol (11.0 mg) of Zn(OAc)_3_^.^2H_2_O in the initial mixture in order to study the characteristics of Zn-Ag-In-Te solid solution NCs. This corresponds to Zn:Ag ratios of 0.5:1 and 1:1, respectively, and after 120 min of reaction, both samples contained NCs with similar sizes. Indeed, the size distributions measured from the TEM images in Fig. 4a and b are 9 (σ = ±2) nm and 11 (σ = ±2) nm for Zn:Ag ratios of 0.5:1 and 1:1 respectively. We recorded PL spectra centered between 1045 nm and 1095 nm in the former case and between 940 nm and 1005 nm in the latter case, as presented in Fig. 4c. The complete series of PL spectra as a function of reaction time is provided in the supporting information (Additional file 1: Figure S4). The XRD diffractograms recorded for both samples were almost identical to those of AIT NCs (Additional file 1: Figure S5), without any signature of other inorganic compounds after purification. The PL QY relative to ICG was evaluated for both samples. The expected increase in radiative emission for these Zn-Ag-In-Te NCs was observed with a PL QY of 2.7 % for a Zn:Ag ratio of 0.5:1 and of 3.4 % for a Zn:Ag ratio of 1:1 after 180 min of reaction. However, the PL was still quickly quenched when the NCs were exposed to air.

## Conclusions

In summary, we prepared for the first time near-stoichiometric AgInTe_2_ NCs emitting in the NIR via a versatile route involving thermolysis of an Ag-In-thiolate complex and progressive incorporation of In^3+^. When Zn(OAc)_2_ was added at the beginning of the synthesis, a significant increase in PL QY up to 3.4 % was observed for a Zn:Ag ratio of 1:1. In all instances, the NCs were quickly oxidized inducing PL quenching after 1 day of storage. Therefore, further work will be needed in order to increase air stability of AgInTe_2_ and Zn-Ag-In-Te NCs.

## Additional file

Additional file 1:
**Supporting information.** The file contains Chemicals, Equipment, Analysis methods and Figures S1 to S5.

## Abbreviations

NCs: Nanocrystals
cQDs: Colloidal quantum dots
TBP: Tributylphosphine
AIT: AgInTe_2_
DDT: Dodecanethiol
ODE: Octadecene
OLA: Oleylamine
TOP: Trioctylphosphine
TCE: Tetrachloroethylene
PL: Photoluminescence
EDS: Energy-dispersive X-ray spectroscopy
TEM: Transmission electron microscopy
XRD: X-ray diffraction
QY: Quantum yield
ICG: Indocyanine green
