# Supplementary material for: Near-infrared emitting AgInTe2 and Zn-Ag-In-Te colloidal nanocrystals
Source: Nanoscale Res Lett. 2015 Jun 5;10:255. doi: 10.1186/s11671-015-0951-y (PMC4477004; doi:10.1186/s11671-015-0951-y)
Supplement: Additional file 1: — Supporting information. The file contains Chemicals, Equipment, Analysis methods and Figures S1 to S5. [file 11671_2015_951_MOESM1_ESM.pdf]

Near-Infrared Emitting $AgInTe_2$ and Zn-Ag-In-Te Colloidal Nanocrystals

Marc-Antoine Langevin[a.b], Thomas Pons[c], Anna M. Ritcey[a.b], Claudine Nì. Allen[b]

[a]Centre de Recherche sur les Matériaux Avancés (CERMA), Département de Chimie, Université Laval, 1045 Av. de la Médecine, Québec (Québec), Canada, G1V 0A6.

[b]Centre d'Optique, Photonique et Laser (COPL), Département de Physique, de Génie Physique et d'Optique, Université Laval, 2375 Rue de la Terrasse, Québec (Québec), Canada, G1V 0A6.

[c] Laboratoire Physique et Étude des Matériaux, ESPCI/CNRS/UPMC UMR8213, 10 Rue Vauquelin, 75005 Paris, France.

Supporting information

Chemicals Silver(I) oxide (Ag_2O, 99+% metal basis, Alfa Aesar); indium(III) acetate ($In(OAc)_3$, anhydrous 99.99% trace metal basis, Acros Organics);1-dodecanethiol (DDT, 98% Alfa Aesar);1-octadecene (ODE, technical grade 90%, Alfa Aesar); oleylamine (OLA, technical grade 70%, Sigma Aldrich); tellurium powder (Te, ~200 mesh, 99.8% metal basis, Sigma Aldrich); tributylphosphine (TBP, 97%, Sigma Aldrich); trioctylphosphine (TOP, min 97%, Strem Chemicals); zinc acetate dihydrate ($Zn(OAc)_2 \cdot 2H_2O$, 97+%, Alfa Aesar); tetrachloroehylene (TCE, technical grade, Anachemia); methanol (Certified ACS grade, Fisher Chemicals); anhydrous ethanol (ACS grade, Commercial Alcohols); 2-propanol (Certified ACS, Fisher Chemicals); acetone (ACS grade, CFS Chemicals); dimethylsulfoxide (DMSO, Certified ACS, Fisher Chemicals); indocyanine green (ICG, Cardiogreen, Sigma Aldrich).

Equipment and analysis method

Absorption spectra of the AIT NCs in TCE and the reference fluorophore were acquired with a Varian Cary 50 Conc UV-visible spectrophotometer. The suspensions were kept diluted enough to avoid self-absorption.

Photoluminescence spectra were recorded between 800 nm and 2000 nm with a Jobin Yvon Nanolog equipped with a Symphony-II CCD detector. The excitation wavelength was set to 488 nm for $AgInTe_2$ and Zn-Ag-In-Te NCs dispersed in TCE.

Fluorescence quantum yield (PL QY) was determined as presented by Williams *et al.* [*1*]. First, UV-visible absorption spectra were acquired for the indocyanine green (ICG) reference at four different concentrations. Measurements were taken at low concentrations in order to avoid reabsorption that would result in nonlinear effects affecting the calculated PL QY values. PL spectra were then recorded for each dilution and the integrated PL intensity (in photons) was plotted as a function of absorbance at λ_{abs} = 795 nm for ICG and λ_{abs} = 488 nm for AIT and Zn-Ag-In-Te NCs. PL QY was calculated with the following equation:

$$QY_{NCs} = QY_{ICG}\left(\frac{Grad_{NCs}}{Grad_{ICG}}\right)\left(\frac{\eta_{TCE}^2}{\eta_{DMSO}^2}\right)$$

where *Grad* is the gradient of the data plotted and η is the refractive index of the solvent.

Energy-dispersive x-ray spectroscopy measurements were obtained with a Quanta 3D FEG scanning electron microscope from the FEI Company using an EDAX Si(Li) detecting unit. Drops of well purified AIT NCs were put on a carbon surface and scanned with a 15 kV electron beam.

Powder X-ray diffractograms were acquired using a Siemens/Bruker X-ray diffractometer with a 2D Hi-Star XRD detector. The radiation source was a Kristalloflex 760 with a nickel window

emitting the Cu K_α line (λ = 1.5418 Å) with an accelerating voltage and current of 40 kV and 40 mA respectively. Diffractograms were recorded from 10° to 60°. The background signal was automatically subtracted by the diffraction pattern treatment software GADDS.

Transmission electron microscopy images were obtained with a JEOL JEM 1230. For each sample, 200 NCs were measured by fitting circles around NCs and extracting their diameter using an image treatment software (ImageJ). Slightly elongated NCs were also considered as spheres. Most of the elongated NCs are actually composed of close smaller spherical NCs that were considered independently for the determination of the average size. Considering this, only a minority of larger elongated NCs were not considered for the determination of the average size.

References

[1] Williams ATR, Winfield SA, Miller JN. Relative Fluorescence Quantum Yields Using a Computer Controlled Luminescence Spectrometer. Analyst. 1983;108:1067-71.

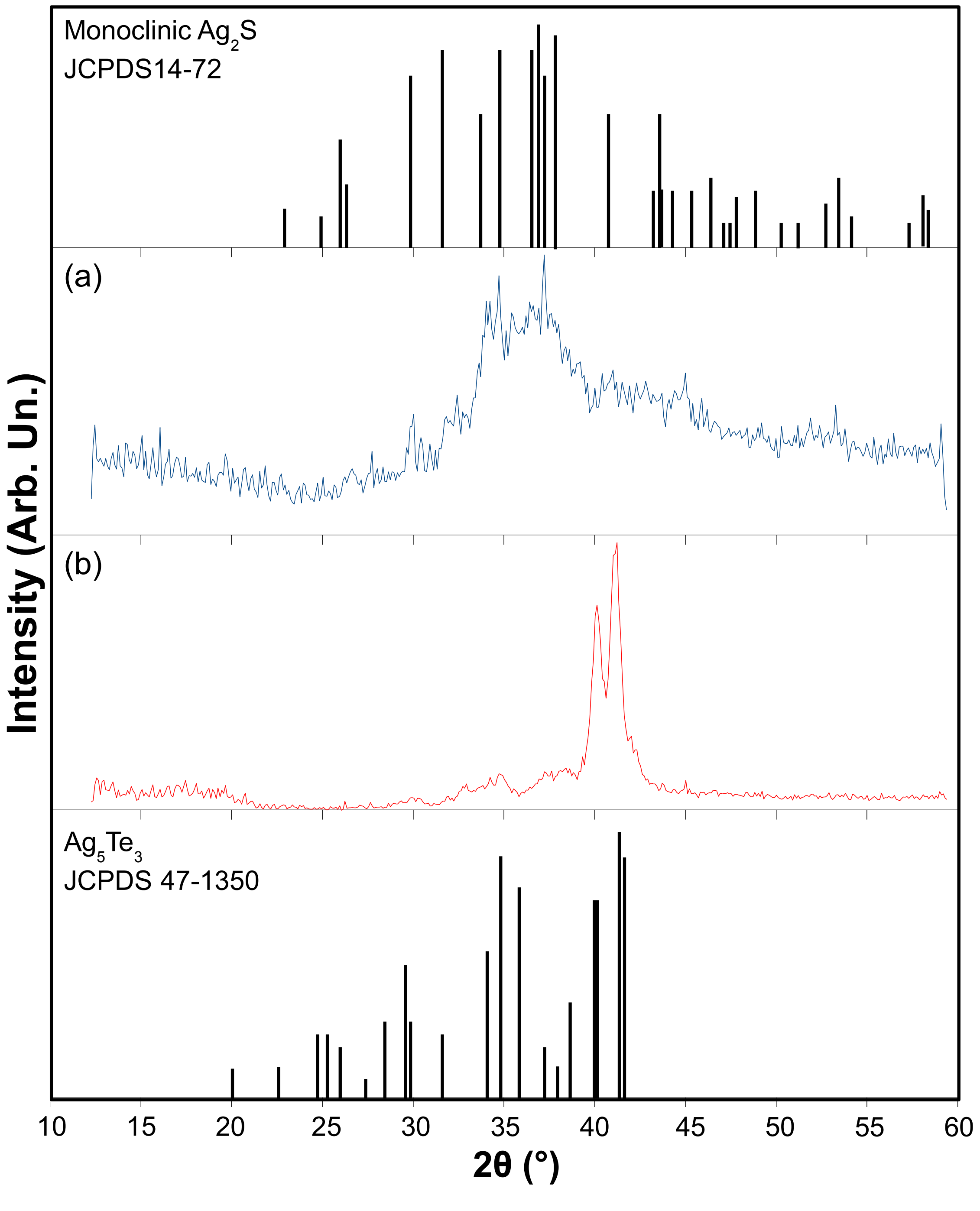

<u>Figure S1</u>. XRD pattern of (a) ~4 nm Ag_2S NCs synthesized using Zhang *et al.* method and (b) resulting Ag_2Te obtained immediately after injection of TOP-Te in a suspension of ~4 nm Ag_2S NCs dispersed in ODE with OLA and DDT at 170°C.

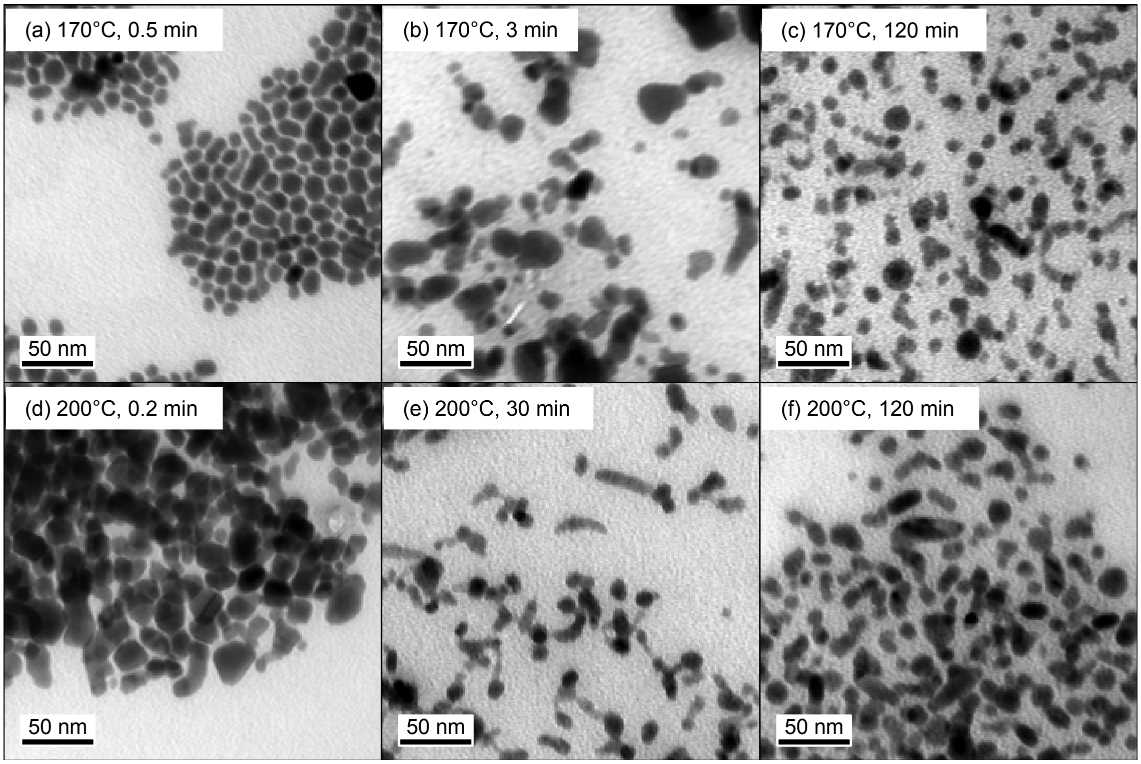

Figure S2. TEM images of AIT NCs synthesized at 170°C after (a) 0.5 min, 10.6 ($\sigma=\pm3$) nm (b) 3 min,15 ($\sigma=\pm6$) nm (c) 120 min, 10.7 ($\sigma=\pm3$) nm and at 200°C after (d) 0.2 min, 13 ($\sigma=\pm4$) nm (e) 30 min, 10.3 ($\sigma=\pm2$) nm (f) 120 min, 12.8 ($\sigma=\pm2$) nm.

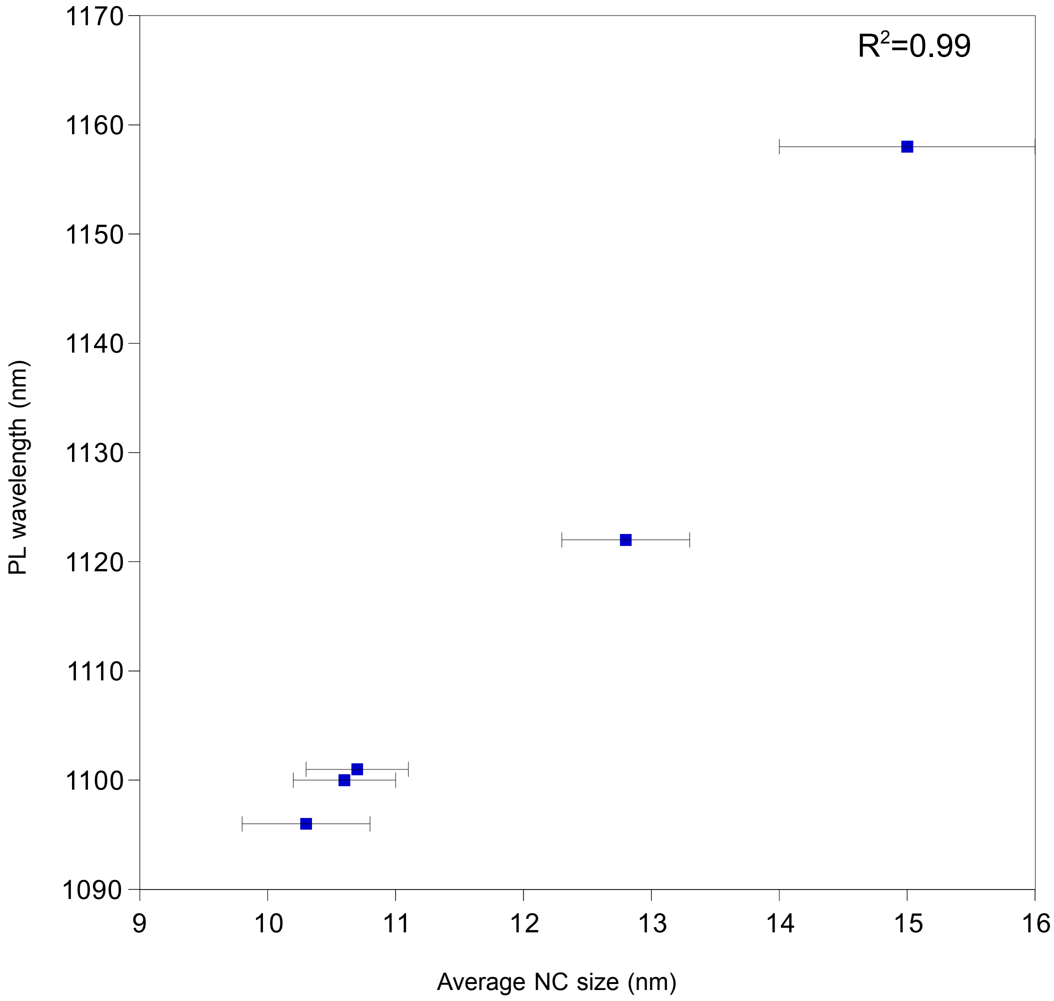

Figure S3. PL emission wavelength as a function of the average NC size determined by TEM. The error bars represent the uncertainty on the average NC size.

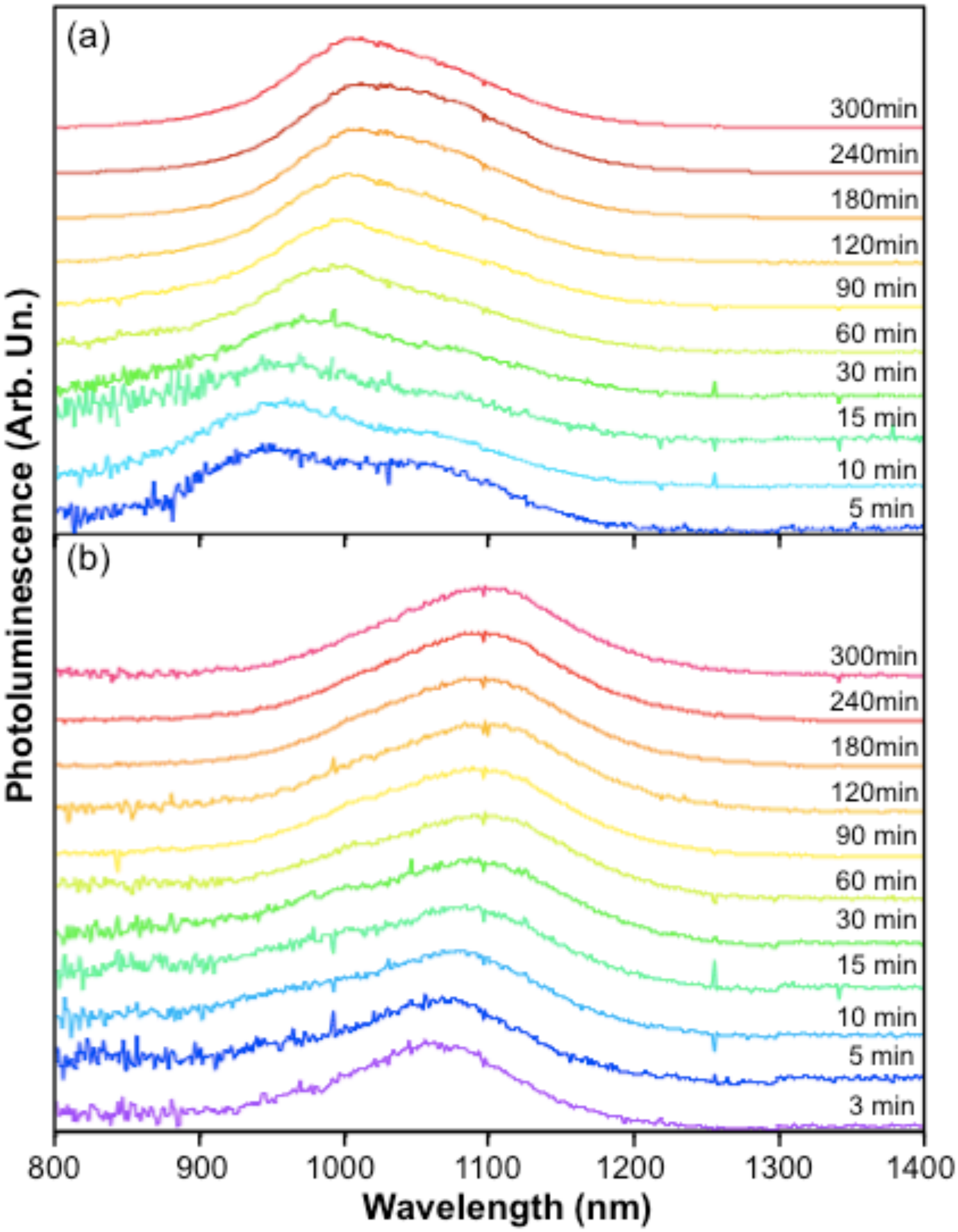

Figure S4. Photoluminescence spectra of Zn-Ag-In-Te nanocrystals with a Zn:Ag ratio of (a) 1:1 and (b) 0.5:1.

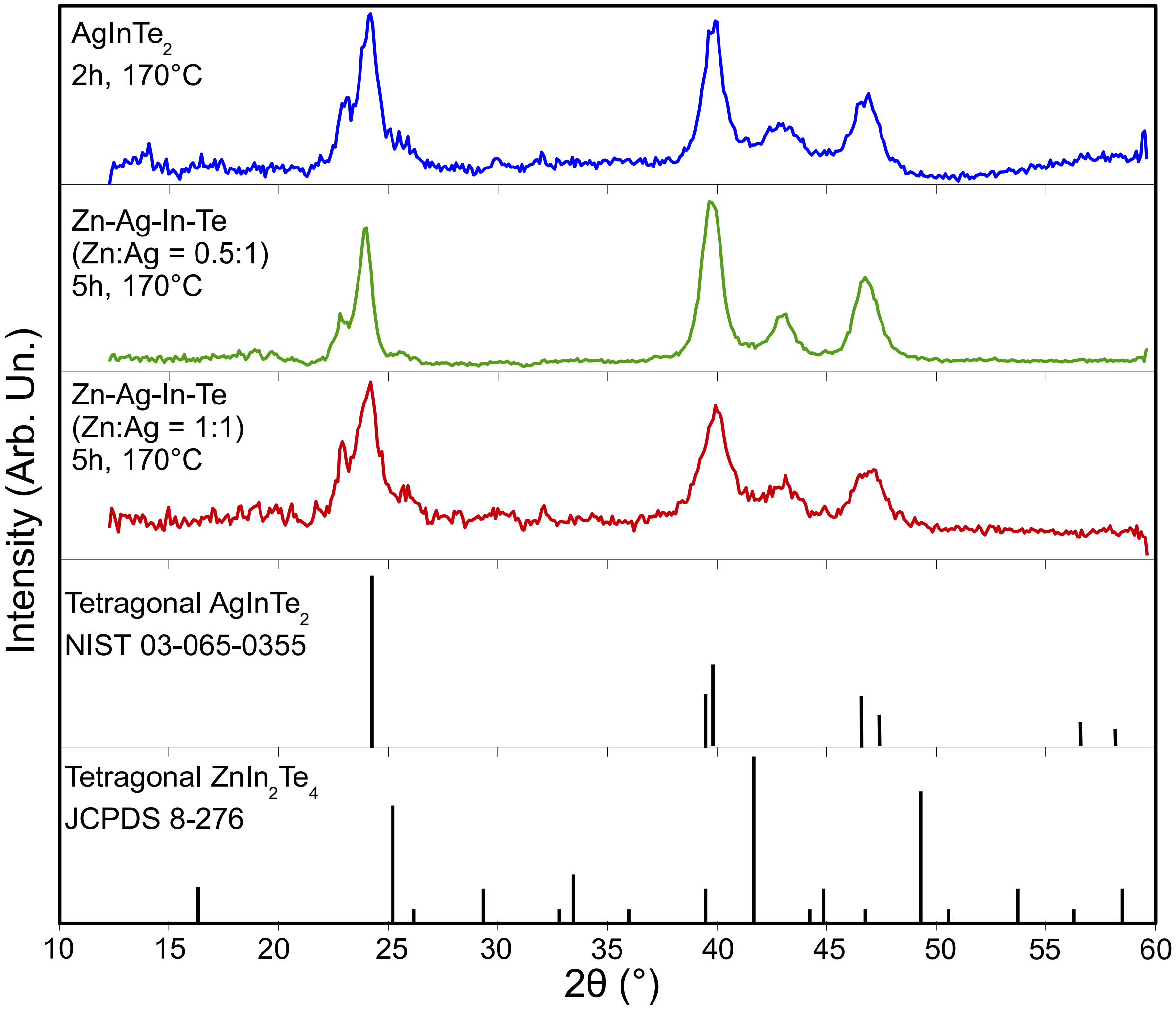

Figure S5. XRD diffractograms of AIT (blue) and ZAIT with a Zn:Ag ratio of 0.5:1 (green) and 1:1 (red)
